# The complete genome sequence of the yogurt isolate *Streptococcus thermophilus* ACA-DC 2

**DOI:** 10.1186/s40793-017-0227-5

**Published:** 2017-01-31

**Authors:** Voula Alexandraki, Maria Kazou, Jochen Blom, Bruno Pot, Effie Tsakalidou, Konstantinos Papadimitriou

**Affiliations:** 1 Laboratory of Dairy Research, Department of Food Science and Human Nutrition, Agricultural University of Athens, Iera Odos 75, 118 55 Athens, Greece; 2 Bioinformatics & Systems Biology, Justus-Liebig-University Giessen, 35392 Giessen, Hesse Germany; 3 Research Group of Industrial Microbiology and Food Biotechnology (IMDO), Vrije Universiteit Brussel, Brussels, Belgium

**Keywords:** Extended genome report, *Streptococcus thermophilus*, Yogurt, Horizontal gene transfer, CRISPR, Stress genes

## Abstract

*Streptococcus thermophilus* ACA-DC 2 is a newly sequenced strain isolated from traditional Greek yogurt. Among the 14 fully sequenced strains of *S. thermophilus* currently deposited in the NCBI database, the ACA-DC 2 strain has the smallest chromosome, containing 1,731,838 bp. The annotation of its genome revealed the presence of 1,850 genes, including 1,556 protein-coding genes, 70 RNA genes and 224 potential pseudogenes. A large number of pseudogenes were identified. This was also accompanied by the absence of pathogenic features suggesting evolution of strain ACA-DC 2 through genome decay processes, most probably due to adaptation to the milk ecosystem. Analysis revealed the existence of one complete lactose-galactose operon, several proteolytic enzymes, one exopolysaccharide cluster, stress response genes and four putative antimicrobial peptides. Interestingly, one CRISPR-cas system and one orphan CRISPR, both carrying only one spacer, were predicted indicating low activity or inactivation of the cas proteins. Nevertheless, four putative restriction-modification systems were determined that may compensate any deficiencies of the CRISPR-cas system. Furthermore, whole genome phylogeny indicated three distinct clades within *S. thermophilus*. Comparative analysis among selected strains representative for each clade, including strain ACA-DC 2, revealed a high degree of conservation at the genomic scale, but also strain specific regions. Unique genes and genomic islands of strain ACA-DC 2 contained a number of genes potentially acquired through horizontal gene transfer events, that could be related to important technological properties for dairy starters. Our study suggests genomic traits in strain ACA-DC 2 compatible to the production of dairy fermented foods.

## Introduction

The use of microorganisms in food fermentations is the means for converting perishable and frequently inedible raw materials into safe, shelf-stable and nutritionally upgraded foods [1]. The economic importance of starter cultures for the food industry has led to the continuous search for the discovery of new microorganisms with important technological characteristics. In many cases it has been proven that traditionally fermented foods represent a natural reservoir of undiscovered microbial strains for possible diverse food applications [2, 3].

*Streptococcus thermophilus* is among the species commonly used in the dairy industry, mainly in the fermentation of yogurt and several cheese varieties, contributing to the desirable organoleptic characteristics of the final product [4, 5]. It is the sole species considered GRAS within the *Streptococcus* genus, which includes mostly pathogens and opportunistic pathogens [6]. Due to the industrial significance of the species, a plethora of studies has been conducted for a number of strains, revealing information about their diverse technological features [7, 8]. Furthermore, during the last 15 years, the advance of high-throughput sequencing techniques along with the development of novel bioinformatics tools facilitated the analysis of complete genome sequences, providing information for the overall genetic content of *S. thermophilus* [9–12]. These studies have demonstrated that *S. thermophilus* strains have been adapted to the milk environment through extensive reductive evolution as indicated by the large number of pseudogenes found in all strains. Adaptation to the milk environment is also supported by the loss of genes related to carbohydrate metabolism and virulence.

In this study, we present the analysis of the complete genome sequence of *S. thermophilus* ACA-DC 2. The genomic insights acquired could be proven useful for the exploitation of the specific strain in the production of fermented dairy products.

## Organism information

### Classification and features

**Fig. 1:**
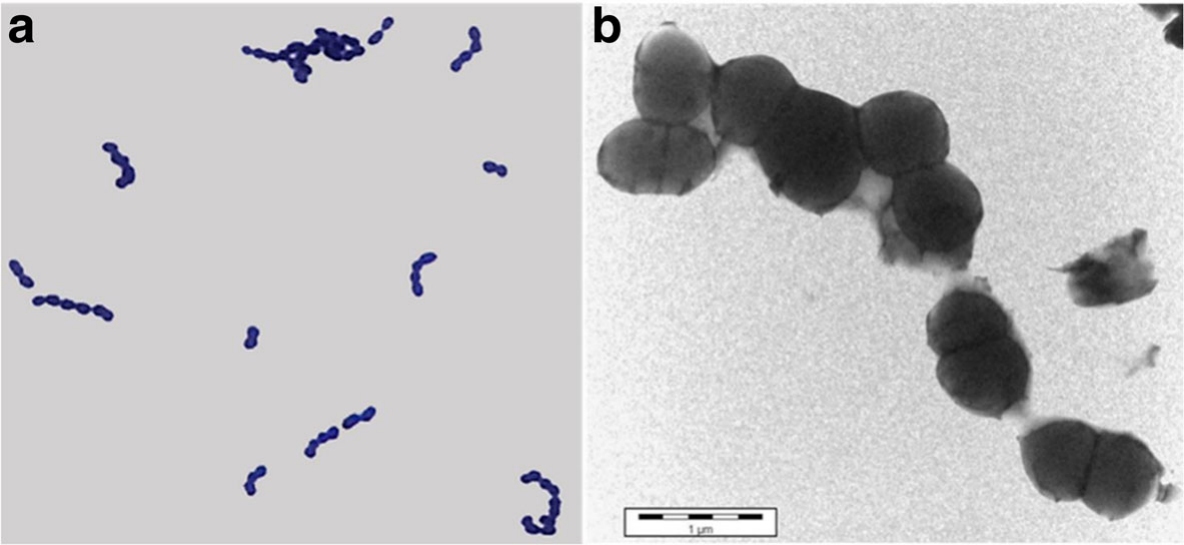
Photomicrographs of *S. thermophilus* ACA-DC 2. The images were obtained with (**a**) optical microscopy at magnification 1000x for Gram stained cells and (**b**) transmission electron microscopy of stained cells with 10% (w/v) PTA. Scale bar in (**b**) corresponds to 1 μm

**Fig. 2:**
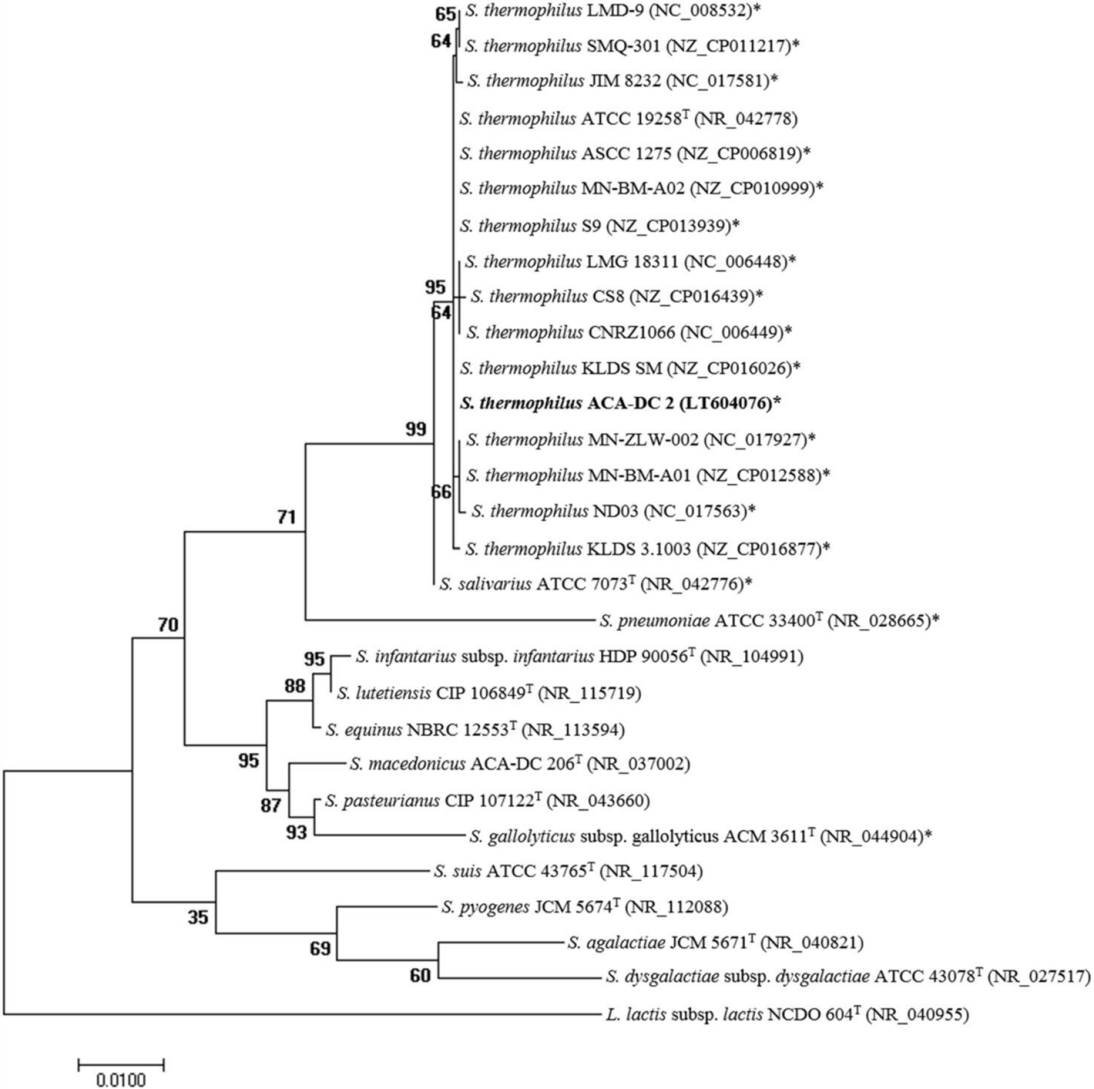
Phylogenetic tree highlighting the position of *S. thermophilus* ACA-DC 2 relative to other *Streptococcus* species. The tree was constructed based on 16S rRNA gene sequences. GenBank accession numbers are presented in parentheses and type strains are indicated with a superscript T (type strains = ^T^). Strains with complete genome sequence are marked with an asterisk. 16S rRNA gene sequences were aligned using MUSCLE [55]. The phylogenetic tree was built by the Maximum Likelihood method within MEGA7 software [56] using the Tamura-Nei substitution model [57]. *Lactococcus lactis* subsp. *lactis* NCDO 604^T^ served as the outgroup. Bootstrap values derived after 1,000 replicates. The scale bar indicates an estimated 0.01 nucleotide change per nucleotide position

*Streptococcus thermophilus* ACA-DC 2 is classified within the order *Lactobacillales* of the class *Bacilli*. It is a non-sporulating, Gram-positive bacterium with coccus-shaped cells (Fig. 1). The strain was isolated from traditional Greek yogurt manufactured through backslopping [13, 14]. Its optimum growth takes place in M17 medium at 42 ^o^C under microaerophilic conditions within 24 h. Information about the classification and the features of *S. thermophilus* ACA-DC 2 is summarized in Table 1. The phylogenetic analysis was based on 16S rRNA gene sequences and places *S. thermophilus* ACA-DC 2 in the distinct cluster formed by the *S. thermophilus* strains and within the salivarius group, as shown in Fig. 2.

**Table 1:** Classification and general features of *S*. *thermophilus* strain ACA-DC 2 according to the MIGS recommendations [39]

MIGS ID	Property	Term	Evidence code^a^
	Classification	Domain *Bacteria*	TAS [40]
		Phylum *Firmicutes*	TAS [41, 42]
		Class *Bacilli*	TAS [43, 44]
		Order *Lactobacillales*	TAS [44, 45]
		Family *Streptococcacceae*	TAS [46–48]
		Genus *Streptococcus*	TAS [47, 49, 50]
		Species *Streptococcus thermophilus*	TAS [47, 51, 52]
		Strain: ACA-DC 2	TAS (this study)
	Gram stain	Positive	IDA
	Cell shape	Coccus	IDA
	Motility	Non-motile	IDA
	Sporulation	Non-sporulating	NAS
	Temperature range	30–50 °C	TAS [51]
	Optimum temperature	42 °C	TAS [53]
	pH range; Optimum	5–7; 6.5	TAS [53]
	Carbon source	lactose; saccharose; d-glucose; galactose	IDA
MIGS-6	Habitat	Yogurt	TAS [13, 14]
MIGS-6.3	Salinity	2% NaCl (w/v)	TAS [51]
MIGS-22	Oxygen requirement	Microaerophilic	TAS [51]
MIGS-15	Biotic relationship	Free-living	NAS
MIGS-14	Pathogenicity	Non-pathogen	NAS
MIGS-4	Geographic location	Greece	TAS [13, 14]
MIGS-5	Sample collection	1988	NAS
MIGS-4.1	Latitude	Unknown	
MIGS-4.2	Longitude	Unknown	
MIGS-4.4	Altitude	Unknown	

^a^Evidence codes - *IDA* inferred from direct assay, *TAS* traceable author statement (i.e., a direct report exists in the literature), *NAS* non-traceable author statement (i.e., not directly observed for the living, isolated sample, but based on a generally accepted property for the species, or anecdotal evidence). These evidence codes are from the Gene Ontology project [54]

## Genome sequencing information

### Genome project history

*S. thermophilus* ACA-DC 2 is deposited in the ACA-DC culture collection of the Laboratory of Dairy Research, Agricultural University of Athens, Athens, Greece. The strain was selected for sequencing in order to obtain information about its technological and probiotic potential, having as basic aim its application as a starter culture in the production of dairy fermented foods. The project was carried out in 2015 and the genome was sequenced, fully assembled and annotated. The genome sequencing project was registered in the European Nucleotide Archive database under accession number LT604076. The summary of the project information is shown in Table 2.

**Table 2:** Project information

MIGS ID	Property	Term
MIGS 31	Finishing quality	Finished
MIGS-28	Libraries used	Illumina genomic Nextera XT library; PacBio 10 kb genomic library
MIGS 29	Sequencing platforms	Illumina HiSeq2500; PacBio RSII
MIGS 31.2	Fold coverage	636x
MIGS 30	Assemblers	ABySS v1.5.1; BLASR; SSPACE v1.0; GapFiller v1.10
MIGS 32	Gene calling method	Prodigal; MeteGeneAnnotator; FGENESB
	Locus Tag	STACADC2
	Genbank ID	LT604076
	GenBank Date of Release	29-Jul-2016
	GOLD ID	NA
	BIOPROJECT	PRJEB14916
MIGS 13	Source Material Identifier	ACA-DC 2
	Project relevance	Dairy isolate

### Growth conditions and genomic DNA preparation

*S. thermophilus* ACA-DC 2 was grown in M17 medium (Biokar Diagnostics, Beauvais, France). For the isolation of the genomic DNA, 2 ml from an overnight culture incubated at 42 °C were used and the extraction procedure was performed according to the protocol of Pitcher et al. [15]. The purity and the concentration of the extracted DNA were measured with a UV-Vis spectrophotometer (Q5000, Quawell, San Jose, USA) while its integrity was evaluated electrophoretically in a 0.8% agarose gel.

### Genome sequencing and assembly

Whole-genome sequencing was performed using the Illumina HiSeq2500 and the PacBio RSII platforms at BaseClear service laboratory for DNA-research (Leiden, The Netherlands). Paired-end sequence reads were generated using the Illumina HiSeq2500 system. FASTQ sequence files were obtained using the Illumina Casava pipeline v1.8.3. Initial quality assessment was based on data passing the Illumina Chastity filtering. Subsequently, reads containing adapters and/or PhiX control signal were removed using an in-house filtering protocol. The second quality assessment was based on the remaining reads using the FASTQC quality control tool v0.10.0 resulting in 4,403,680 reads.

The data collected from the PacBio RSII instrument were processed and filtered using the SMRT Analysis software suite. The Continuous Long Read data were filtered by Read-length (>50), Subread-length (>50) and Read quality (>0.75) resulting in 117,020 reads.

The quality of the Illumina FASTQ sequences was enhanced by trimming off low-quality bases using the program bbduk, which is part of the BBMap suite v34.46. The quality-filtered sequence reads were puzzled into a number of contig sequences. The analysis was performed using ABySS v1.5.1. The contigs were linked and placed into super-scaffolds based on the alignment of the PacBio CLR reads with BLASR [16]. The alignment was further used to estimate the orientation, order and distance between the contigs by the SSPACE-LongRead scaffolder v1.0 [17]. The gapped regions within the super-scaffolds were closed in an automated manner using GapFiller v1.10 [18]. The method takes advantage of the insert size between the Illumina paired-end reads. The assembly resulted in one circular chromosome of 1,731,838 bp.

### Genome annotation

Prediction of genes was carried out with the online programs Prodigal [19], MetaGeneAnnotator [20] and FGENESB [21], for comparison and verification of the obtained results. Genome annotation was performed using RAST v2.0 [22]. Annotation anomalies, including pseudogenes, were identified using GenePRIMP [23]. All data acquired were combined and subjected to manual curation. The WebMGA server [24] and the EggNog v4.5 [25] were used for COG annotation, the Phobius web server was used for the identification of genes with transmembrane helices and genes with signal peptides [26] and the Pfam database was used for the identification of genes with Pfam domains [27]. Potential pathogenic features were identified using the MP3 tool [28]. The CRISPRs﻿, the restriction-modification systems and the﻿ putative antimicrobial peptides were predicted using the CRISPRFinder web tool [29], the REBASE database [30] and BAGEL3 [31], respectively. The KODON software (Applied Maths NV, Sint-Martens-Latem, Belgium) was utilized for the visualization of synteny among the CRISPR regions of ACA-DC 2 and LMD-9 strains. The EDGAR server [32] was used for whole genome phylogeny and Venn diagram analysis. Circoletto [33] was employed for whole genome alignment among *S. thermophilus* strains. Finally, the genomic islands were identified through the IslandViewer 3 web-based resource [34].

## Genome properties

**Fig. 3:**
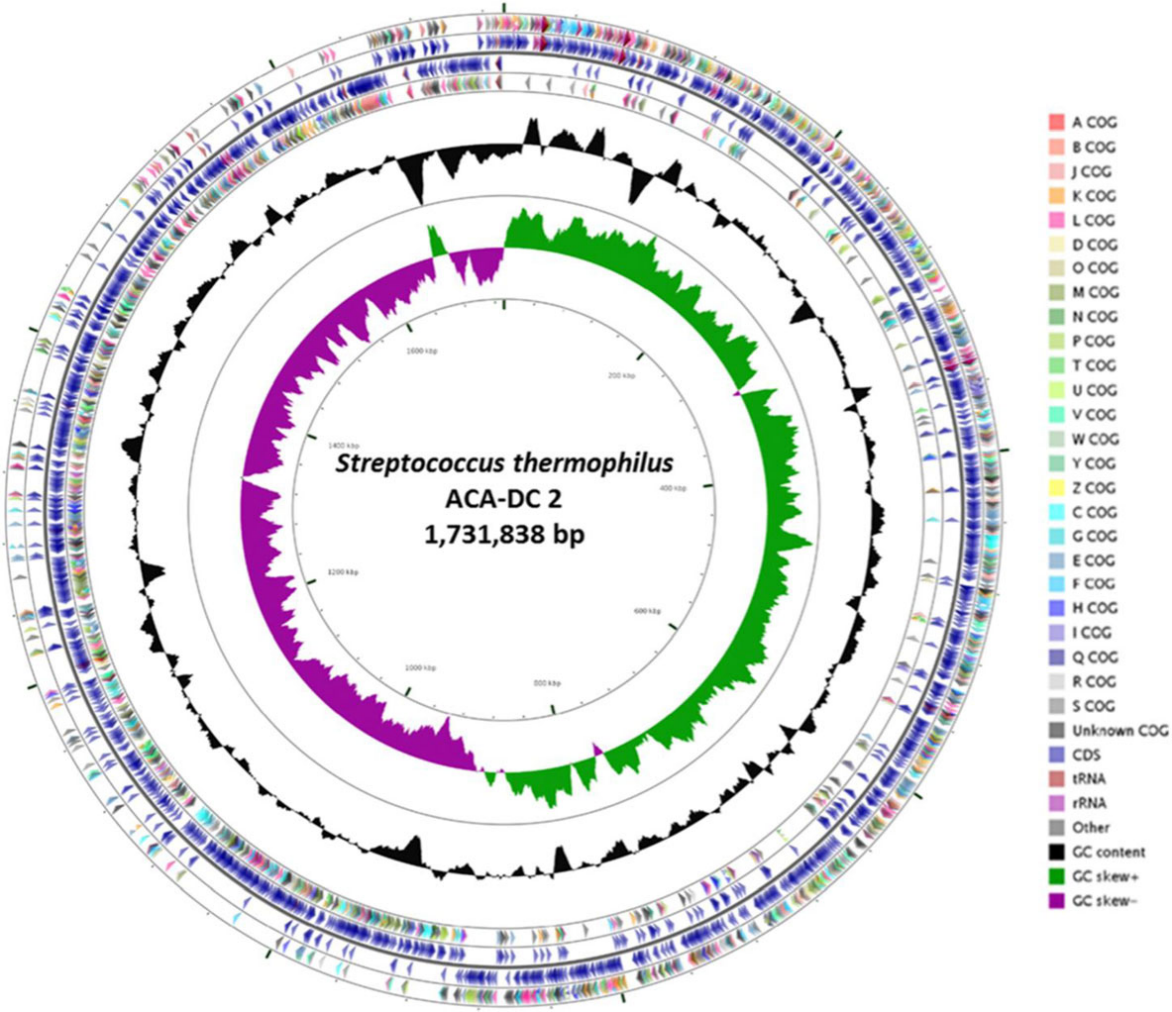
Circular map of *S. thermophilus* ACA-DC 2 genome features generated with the CGview tool. From periphery to center: Protein coding genes on forward strand colored by COG category assignment; Genes on forward strand; Protein coding genes on reverse strand colored by COG category assignment; Genes on reverse strand; GC content; GC skew; Genome region in kbp

The complete genome of *S. thermophilus* ACA-DC 2 consists of one circular chromosome containing 1,731,838 bp. The average GC content of the chromosome is 39.2%. A total of 1,850 genes were predicted after manual curation, including 1,556 protein-coding genes, 70 RNAs (56 tRNAs and 14 rRNAs) and 224 potential pseudogenes (Table 3). A circular map of the genome was generated using the CGView comparison tool [35] as shown in Fig. 3. Function was assigned to 1,182 genes (63.89%), while 1,318 genes (71.24%) had one or more conserved Pfam domains. The distribution of protein-coding genes into COG functional category is shown in Table 4. The analysis revealed that approximately 28.5% of the protein-coding genes do not have any described function.

**Table 3:** Genome statistics

Attribute	Value	% of Total
Genome size (bp)	1,731,838	100.00
DNA coding (bp)	1,356,670	78.34
DNA G + C (bp)	679,104	39.21
DNA scaffolds	1	100.00
Total genes	1,850	100.00
Protein coding genes	1,556	84.11
RNA genes	70	3.78
Pseudo genes	224	12.11
Genes in internal clusters	NA	NA
Genes with function prediction	1,182	63.89
Genes assigned to COGs	1,327	71.73
Genes with Pfam domains	1,318	71.24
Genes with signal peptides	127	6.86
Genes with transmembrane helices	339	18.32
CRISPR repeats	2

**Table 4:** Number of genes associated with general COG functional categories

Code	Value	%age	Description
J	146	9.38	Translation, ribosomal structure and biogenesis
A	0	0.00	RNA processing and modification
K	89	5.72	Transcription
L	136	8.74	Replication, recombination and repair
B	0	0.00	Chromatin structure and dynamics
D	16	1.03	Cell cycle control, Cell division, chromosome partitioning
V	39	2.51	Defense mechanisms
T	43	2.76	Signal transduction mechanisms
M	80	5.14	Cell wall/membrane biogenesis
N	3	0.19	Cell motility
U	20	1.29	Intracellular trafficking and secretion
O	55	3.53	Posttranslational modification, protein turnover, chaperones
C	40	2.57	Energy production and conversion
G	66	4.24	Carbohydrate transport and metabolism
E	160	10.28	Amino acid transport and metabolism
F	67	4.31	Nucleotide transport and metabolism
H	49	3.15	Coenzyme transport and metabolism
I	33	2.12	Lipid transport and metabolism
P	67	4.31	Inorganic ion transport and metabolism
Q	13	0.84	Secondary metabolites biosynthesis, transport and catabolism
R	63	4.05	General function prediction only
S	215	13.82	Function unknown
-	229	14.72	Not in COGs

The total is based on the total number of protein coding genes in the genome

## Insights from the genome sequence

### Main genome sequence characteristics

The genome of *S. thermophilus* ACA-DC 2 is the smallest one described to date among the fully sequenced strains of the species deposited in NCBI and it is approximately 200 kbp smaller than the larger described genome. The majority of potential pseudogenes encode hypothetical proteins, transposases and proteins involved in carbohydrate transport and metabolism. Analysis of the genome for virulence factors with the MP3 tool revealed a number of hits (data not shown). Detailed inspection of these hits with EDGAR demonstrated that several such genes are conserved among *S. thermophilus* strains indicating that it is rather unlikely to be related to pathogenicity, given the safe history of the species. The high percentage of pseudogenes along with the absence of typical virulence factors for streptococci suggest that the ACA-DC 2 strain evolved through genome decay during the adaptation to the rich in nutrients dairy environment [9, 11].

*S. thermophilus* ACA-DC 2 carries a complete lactose-galactose operon containing the *galR*, *galK*, *galT*, *galE*, *galM*, *lacS* and *lacZ* genes (STACADC2_1195-1189) and it is able to ferment lactose and galactose, the latter in a fairly slow rate (data not shown). It has been reported that fermentation of galactose is limited among the strains of *S. thermophilus* [11]. As mentioned above, several genes responsible for the transport and degradation of sugars, such as fructose, maltose and trehalose, have been identified as pseudogenes in the genome of ACA-DC 2, further supporting the specialization of the bacterium to catabolize lactose.

The proteolytic system of *S. thermophilus* ACA-DC 2 consists of several genes encoding aminopeptidases, such as *pepA* (STACADC2_1626), *pepC* (STACADC2_0202), *pepF* (STACADC2_0406), *pepM* (STACADC2_1333), *pepN* (STACADC2_0892), *pepO* (STACADC2_1656), *pepP* (STACADC2_1520), *pepQ* (STACADC2_0572), *pepS* (STACADC2_0058), *pepT* (STACADC2_0971), *pepV* (STACADC2_0960), and *pepX* (STACADC2_1446), one oligopeptide *opp* ABC transporter (STACADC2_1229-1233), four polar amino acid ABC transporters (STACADC2_0780-0782, STACADC2_0992-0995, STACADC2_1355-1358, STACADC2_1431-1433), two symporters for branched-chain amino acids (STACADC2_0872, STACADC2_1160), and two glutamine ABC transporters (STACADC2_0547-0548, STACADC2_1281-1282). Strain ACA-DC 2 lacks a cell wall-associated proteinase (PrtS). Although this gene may be important for optimal growth of *S. thermophilus* in milk, its absence is of minor significance when co-cultured with a proteolytic * Lactobacillus delbrueckii* subsp. *bulgaricus* strain, since the release of peptides by the latter is beneficial for the growth of *S. thermophilus* [10, 11].

Similar to other dairy bacteria, *S. thermophilus* ACA-DC 2 is able to synthesize exopolysaccharides (EPS) that may confer improved viscosity and texture to yogurt [4]. The EPS cluster is flanked by a *deoD* gene encoding a purine nucleoside phosphorylase (STACADC2_0949) and a pseudogene originally encoding a beta-glucosidase. Four of these genes, namely *epsA* (STACADC2_0948), *epsB* (STACADC2_0947), *epsC* (STACADC2_0946) and *epsD* (STACADC2_0945) are implicated in the regulation, polymerization, chain length and export of the EPS and are conserved among several *S. thermophilus* strains [36].

The genome analysis of strain ACA-DC 2 revealed a number of genes known to be responsive to unfavorable conditions prevailing during industrial applications. Among them we identified conserved heat shock genes like *grp*E, *dna*K, *dna*J (STACADC2_0105-0107) and *gro*ES, *gro*EL (STACADC2_0179-0180), genes encoding Clp proteases (STACADC2_0071, STACADC2_0315, STACADC2_0526, STACADC2_0544, STACADC2_1391), a proton translocating F_0_F_1_-ATPase system (STACADC2_0430-0437) and a P-type calcium pump ATPase (STACADC2_0983). The strain also harbors genes related to oxidative stress, namely a Mn-superoxide dismutase (STACADC2_0657), a glutathione reductase (STACADC2_0362), two thioredoxins (STACADC2_1043, STACADC2_1624), two thioredoxin reductases (STACADC2_1208, STACADC2_1429), a NADH oxidase (STACADC2_1113) and two sulfoxide reductases (STACADC2_1408, STACADC2_1159). Furthermore, the genome carries four putative antimicrobial peptides that need further investigation (STACADC2_0091, STACADC2_1453, STACADC2_1458 and STACADC2_1709).

**Fig. 4:**
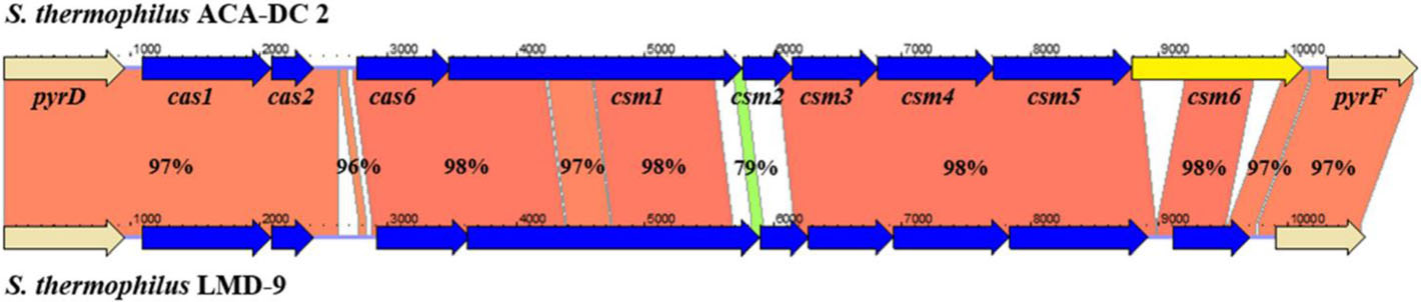
Synteny plot of the CRISPR loci between *S. thermophilus* strains ACA-DC 2 and LMD-9. The synteny of the two regions was calculated by the KODON software. In both strains the *cas* genes are denoted in blue. Gene *csm6* in strain ACA-DC 2 is a potential pseudogene and it is denoted in yellow. The *pyrD* and *pyrF* genes colored in beige define the upstream and downstream limits of the CRISPR loci. Percentages displayed in the ribbon areas correspond to the % identity among the nucleotide sequences

Two candidate CRISPRs were detected in the chromosome of strain ACA-DC 2. Intriguingly, both CRISPRs contained only one spacer. One CRISPR was found surrounded by cas proteins (STACADC2_0849-0856) while the other was orphan. The CRISPR-cas system of strain ACA-DC 2 exhibited the same organization and high degree of identity to that described previously for strain LMD-9 (Fig. 4) [37]. The two CRISPR-cas systems differed mainly in the *csm*6 gene, which in the case of strain ACA-DC 2 is a potential pseudogene as well as in *csm2* gene that seems to be distinct in the two strains. *S. thermophilus* LMD-9 carries three CRISPR-cas systems and the system that is similar to that of ACA-DC 2 carries the lowest number (three) of spacers. Combined these findings could indicate low activity or even inactivation of the entire CRISPR-cas system in strain ACA-DC 2. Another possibility that cannot be excluded concerns low exposure of strain ACA-DC 2 to foreign DNA. Of course, any deficiency in the activity of the CRISPR-cas system may be compensated by restriction-modification (RM) systems. Strain ACA-DC 2 carries four putative RM systems according to the REBASE database (data not shown) belonging to RM types I (STACADC2_0642, STACADC2_0645, STACADC2_0648), II (STACADC2_0597-0598), III (STACADC2_0788-0789) and IV (STACADC2_0626).

### Comparative genomic analysis, strain specific genomic features and genomic islands

**Fig. 5:**
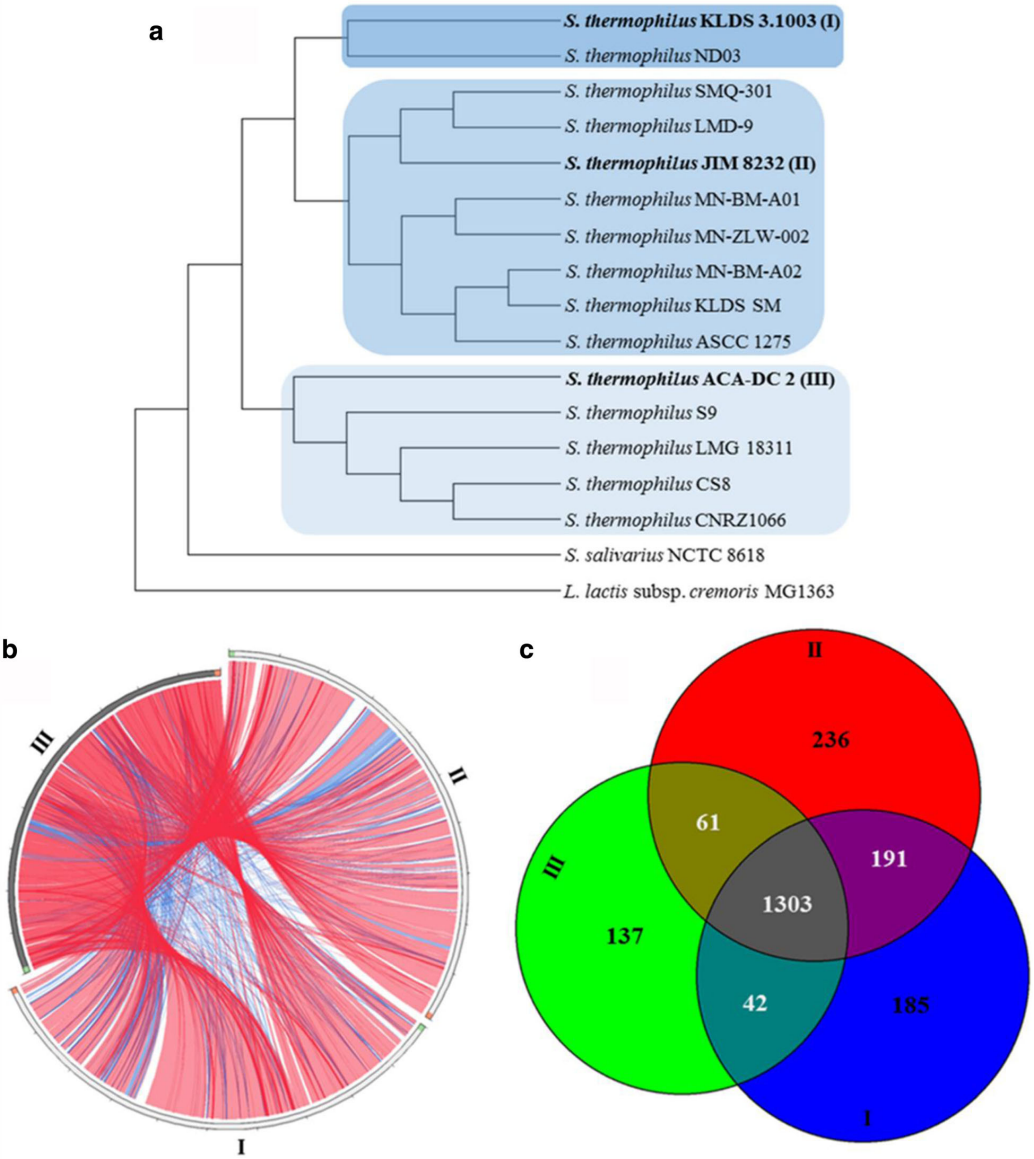
Comparative genomics of *S. thermophilus* strains. **a** Whole genome phylogeny of *S. thermophilus* strains with complete available genome sequences. The phylogenetic tree was calculated in EDGAR and it is presented as a cladogram ignoring branch length. The strains *S. salivarius* NCTC 8616 and *Lactococcus lactis* subsp. *cremoris* MG1363 were used as outgroups. Colored boxes indicate the three distinct *S. thermophilus* branches identified. Strains in each colored branch designated in bold were used for further comparative analysis. **b** Whole genome alignments of *S. thermophilus* strains KLDS 3.1003, JIM 8232 and ACA-DC 2 using Circoletto. Red and blue ribbons correspond to regions of >98% and 80–98% identity, respectively. White regions correspond to strain specific loci. **c** Venn diagram analysis of *S. thermophilus* strains KLDS 3.1003, JIM 8232 and ACA-DC 2, as implemented in EDGAR

Resolution of phylogenetic trees based on 16S rRNA gene sequences is limited due to high sequence identity, especially for strains of the same species. For this reason, we also performed whole genome phylogeny as implemented in EDGAR, using all available complete genomes of *S. thermophilus*. The phylogenetic tree produced revealed that *S. thermophilus* strains could be clustered in two distinct branches, the second of which could be also split in two sub-branches (Fig. 5). Strain ACA-DC 2 formed one of the branches along with strains CNRZ1066, LMG 18311, S9 and CS8. We chose strains ACA-DC 2, JIM 8232 and KLDS 3.1003 as representatives of each branch for comparative genomic analysis (Fig. 5). Whole genome alignments revealed extensive regions of high identity (>98%) among the genomes. However, regions of lower identity (between 80 and 98%) as well as strain specific regions were also identified. Using Venn diagram analysis as implemented in EDGAR, we determined a core genome of 1,303 genes among the three genomes as well as 137, 185 and 236 unique genes for strains ACA-DC 2, KLDS 3.1003 and JIM 8232, respectively.

**Fig. 6:**
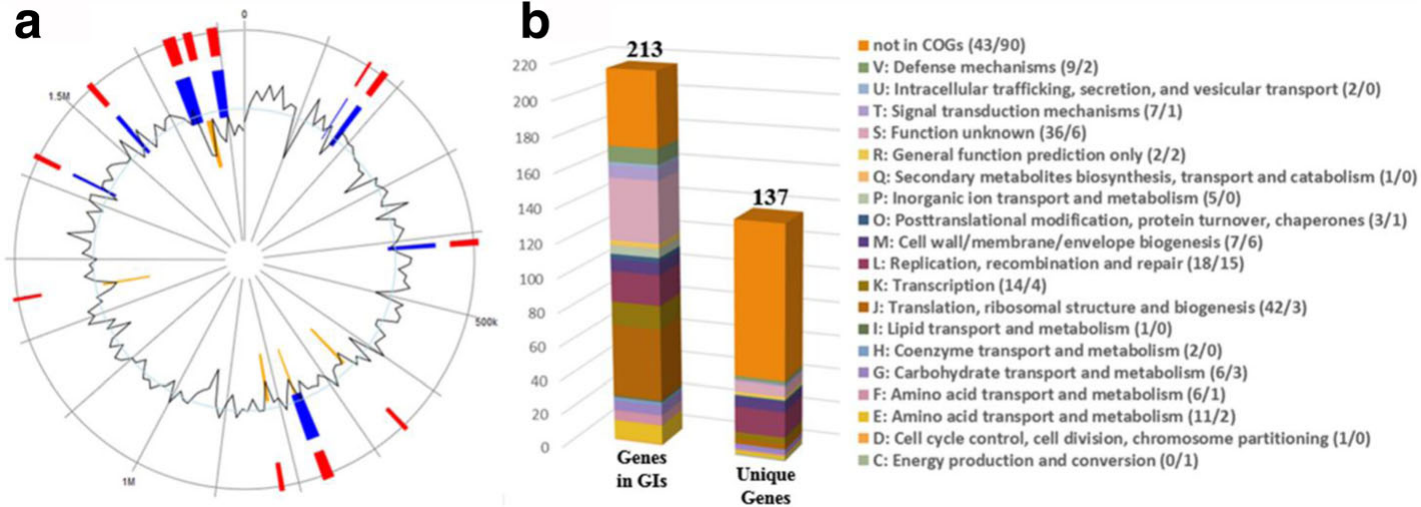
Additional genomic features of *S. thermophilus* ACA-DC 2. **a** Circular map of the *S. thermophilus* ACA-DC 2 genome as generated by IslandViewer 3. Highlighted regions correspond to GIs. GIs are colored within the circular map according to the prediction method used: five GIs in orange and eight GIs in blue were predicted with SIGI-HMM and IslandPath-DIMOB, respectively. Twelve integrated GIs are presented on the periphery in red. The *black* line plot represents the GC content (%) of the genomic sequence. **b** Distribution of genes in GIs and unique genes of *S. thermophilus* ACA-DC 2 into COG categories

The 137 unique genes of strain ACA-DC 2 were found to be involved in diverse functions (Fig. 6). At least some of those genes may be the result of horizontal gene transfer (HGT). HGT acquired genes may play a role in the technological properties of *S. thermophilus* strains [11]. Another analysis that may also reveal regions of HGT in the bacterial chromosome is the identification of GIs [38]. Twelve integrated GIs were predicted in the genome of *S. thermophilus* ACA-DC 2 (Fig. 6), containing a total of 213 genes also involved in diverse functions (Fig. 6). Detailed analysis of genes either unique or in the GIs could relate some of them to important technological traits. For example, we determined genes coding for cold shock proteins CspA and CspG (STACADC2_0749-0750), acid resistance locus arl7 (STACADC2_0743), putative bacteriocin peptides (STACADC2_1453 and STACADC2_1458) and a type I RM system (STACADC2_0642, STACADC2_0645, STACADC2_0648). A putative agmatinase gene (STACADC2_0818) that may play a role to protocooperation of *S. thermophilus* and *L. bulgaricus* during polyamine metabolism, was also detected in ACA-DC 2 strain [10]. Furthermore, genes implicated in zinc and heavy metals transport (STACADC2_0165-0166, STACADC2_0752), in DNA repair and metabolism (STACADC2_1696, STACADC2_1716, STACADC2_1719, STACADC2_1754) as well as several ribosome binding proteins, were also identified (STACADC2_0137, STACADC2_1568-1569, STACADC2_1667, STACADC2_1669-1671, STACADC2_1675-1695, STACADC2_1717, STACADC2_1732-1733, STACADC2_1752, STACADC2_1755).

## Conclusions

The genome of *S. thermophilus* ACA-DC 2 presents characteristics in accordance with its adaptation to the milk environment including a high percentage of pseudogenes and absence of pathogenic features. Our analysis revealed that the strain carries genes involved in lactose and galactose catabolism and protein degradation that may accommodate its growth during milk fermentation. Stress response related genes that may contribute to survival under technological hurdles were also detected. Whole genome phylogeny suggested that *S. thermophilus* strains may diversify in three phylogenetic clades. Comparative analysis of genomes representative of each clade, including strain ACA-DC 2, revealed a number of unique genes for the latter. Furthermore, certain unique genes or genes belonging to GIs could be related to technological properties important for starter cultures. Theoretically, such genes could have been acquired through HGT. These findings render *S. thermophilus* ACA-DC 2 an appropriate candidate for use in the production of fermented dairy products.

## Abbreviations

ACA-DC: Agricultural College of Athens - Dairy Collection
CLR: Continuous long read
COG: Clusters of Orthologous Groups
CRISPR: Clustered regularly interspaced short palindromic repeats
ENA: European nucleotide archive
EPS: Exopolysaccharide
GenePRIMP: Gene prediction improvement pipeline
GI: Genomic Island
GRAS: Generally regarded as safe
HGT: Horizontal gene transfer
PTA: Phosphotungstic acid
RAST: Rapid annotation using subsystem technology
RM: Restriction-modification
SMRT: Single molecule real time
